# Dietary Phytoestrogen Intake and The Risk of Endometriosis in Iranian Women: A Case-Control Study

**DOI:** 10.22074/ijfs.2020.5806

**Published:** 2019-11-11

**Authors:** Samaneh Youseflu, Shahideh Jahanian Sadatmahalleh, Azadeh Mottaghi, Anoshirvan Kazemnejad

**Affiliations:** 1 Department of Reproductive Health and Midwifery, Faculty of Medical Sciences, Tarbiat Modares University, Tehran, Iran; 2 Research Center for Prevention of Cardiovascular Diseases, Institute of Endocrinology and Metabolism, Iran University of Medical Sciences, Tehran, Iran; 3 Department of Biostatistics, Faculty of Medical Sciences, Tarbiat Modares University, Tehran, Iran

**Keywords:** Case-Control Study, Endometriosis, Phytoestrogen

## Abstract

**Background:**

Endometriosis is an important gynecologic disease affecting reproductive-age women. Based on the effect of phytoestrogens on inflammatory, immunological and hormonal factors, limited studies have suggested that phytoestrogen consumption could probably modulate endometriosis risk. The aim of this study was to evaluate the relationship between phytoestrogen intake and endometriosis risk.

**Materials and Methods:**

In the present case-control study, 78 women with a laparoscopically confirmed endome- triosis and 78 normal pelvis women (as the control group), were recruited. Common dietary intake was recorded by a validated 147-item semi-quantitative food frequency questionnaire (FFQ). Type of phytoestrogen in each dietary item was analyzed by the database from the United States Department of Agriculture (USDA). A logistic regression model was used to determine the association between phytoestrogen intake and endometriosis risk.

**Results:**

Higher intake of total phytoestrogen (P-trend=0.01), total isoflavones (P-trend=0.002) specially formononetin (P-trend=0.04) and glycitein (P-trend=0.04), total lignan (P-trend=0.01) specially secoisolariciresinol (P-trend=0.01) and lariciresinol (P-trend=0.02) and matairesinol (P-trend=0.003), and total coumestrol [third quartile odds ratios (OR): 0.38; 95% confidence intervals (CI): 0.15-0.96; P-trend=0.1] was related to reduced endometriosis risk. Among food groups, only isoflavin (OR: 0.48; 95% CI: 0.44-0.63), lignan (OR: 0.66; 95% CI: 0.62-0.94), coumestrol (OR: 0.64; 95% CI: 0.51-0.99), phytoestrogen (OR: 0.46; 95% CI: 0.38-0.83) in dairy products and coumestrol in fruits (OR: 0.69; 95% CI: 0.03-0.77) were negatively associated with endometriosis risk.

**Conclusion:**

Phytoestrogens have a major impact on the level of hormones, and immune and inflammatory markers; thus, it can play an important role in the control and prevention of many diseases. Due to the inflammatory nature of endometriosis and the effect of hormones on the progression of the disease, the role of phytoestrogens consumption in the progression and regression of the disease should be assessed in future works.

## Introduction

Endometriosis is an important gynecologic disease affecting reproductive-age women (1). The prevalence of endometriosis is approximately 10.8 per 1000 women of reproductive age (2). Endometriosis development was shown to be highly related to prolonged exposure to estrogens in the absence of progesterone. The diet has a strong effect on hormonal activity, inflammatory markers and the immune system, therefore, plays an important role in the pathogenesis of endometriosis (3- 5). The results of some studies showed that nutrition and diet have a major impact on endometriosis risk (6- 9). Phytoestrogens are estrogenic components that exist in multiple foods of plant origin. Dietary sources of phytoestrogens have been identified in various food stuffs including fruits, vegetables, spinach, sprouts, beans, cabbage, soybean, grains, and oilseeds (such as flaxseed). Main classes of phytoestrogens consist of isoflavones, coumestans, lignans, and flavonoids. Isoflavones have several subgroups including genistein, daidzein, glycitein, formononetin, biochanin A, and their glycosides. Mammalian lignans include secoisolariciresinol, matairesinol, pinoresinol, lariciresinol, syringaresinol, arctigenin, and 7-hydroxy matairesinol (10). Lignan and isoflavonoid glycosides are converted by gut microflora to hormone-like substances with poor estrogenic activity (0.1% that of estradiol). Therefore, phytoestrogens may demonstrate poor estrogenic activity in low-estrogen environments such as that observed in menopause and have antiestrogenic activity in high-estrogen environments such as that observed in endometriosis or endometrial cancer (11, 12). These substances bind competitively to estrogen receptors, thus blocking endogenous estrogens binding (11). High intake of phytoestrogen is associated with lower C-reactive protein (CRP) concentration (13) and suppressed the immune response (14). 

Phytoestrogens stimulate sex hormone-binding globulin (SHBG) production in the liver (15). High levels of SHBG bind to the free estrogen and diminish the concentration of estrogens available for binding to estrogen receptors (16). 

With regard to the role of inflammatory, immunologic and hormonal factors in the pathogenesis of this disease, we hypothesized that phytoestrogen intake can reduce the risk of endometriosis. Our study evaluated the association between dietary phytoestrogen intake and endometriosis risk using a food frequency questionnaire (FFQ).

## Materials and Methods

Between May 2016 and February 2017, the present case-control study was conducted on 156 infertile women in clinic Arash Hospital, Tehran, Iran. The sample size was determined using the information obtained from a pilot study with 20 patients and the following formula: all infertile women who underwent diagnostic laparoscopy during the period of the study were allocated. The case group consisted of 78 endometriosis women for whom the disease was confirmed by laparoscopy and histology examinations. Control group included 78 infertile women with a normal pelvis. Women in the two groups were comparable in demographic and personal characteristics. 

Inclusion criteria were as follows: i. Age between 15-45 years, ii. The absence of a history of chronic disease (such as cancer, diabetes, stroke, heart disease, etc.), iii. Being from Iranian race, iv. Not being pregnant, v. Not using medications affecting food absorption, appetite and basal metabolism of the body, and vi. No smoking and vii. Lack of mental retardation. 

The medical Ethics Committee of Tarbiat Modares University approved the study (IR.TMU.REC.1395.358) also, before enrolment of the participants, a written informed agreement was obtained from each one. In the beginning, a socio-demographic questionnaire including questions about socioeconomic status, age, smoking, education, habitat, and ethnicity was completed by women, then participants’ dietary information was obtained using FFQ.

### Dietary assessment

Dietary data were collected using FFQ as a validated semi-quantitative questionnaire with 147 food items. Trained dietitians questioned participants regarding their intake frequency for each food item consumed during the past year on a daily, weekly, or monthly basis; all these were converted to daily intakes. Then, by applying the manual for household measures, portion sizes of the consumed food were transformed to grams (17). The validity and reliability of the FFQ for food groups intakes were assessed and were found to be acceptable (18). Type of phytoestrogen per 100 gram of each dietary item was analyzed by the database from the United States Department of Agriculture (USDA) (19) and Tables and databases available from other studies (19-25). Total consumption of phytoestrogen was calculated as the sum of isoflavin, lignan, and coumestrol. We excluded individuals with dissimilar nutrient intake and those with daily energy intake of >4300 or <670 kcal.

### Statistical analysis

Statistical analysis of data was performed by using Statistical Package for Social Science (SPSS, version 21, SPSS Inc., Chicago, IL, USA). Odds ratio [adjusted for age, total energy intake, body mass index (BMI), educational level, and income], with 95% confidence intervals (95% CIs) were calculated using logistic regression models to assess the strength of the associations between the phytoestrogen intake and the risk of endometriosis. Dietary phytoestrogen, isoflavin, lignan, and coumestrol intake was categorized into quartile categories, based on the distribution of control subjects. To calculate the linear trend in the odds of dietary variable quartile, median factor score of each quartile was entered into the logistic regression analysis, and the lowest quartile of intake was used as the reference category for all regression analyses. T test, Mann-Whitney, and chi-square were used to compare other variables. A P value below 0.05 was considered statistically significant.

## Results

Table 1 compares the demographic characteristics of healthy women and subjects with endometriosis. There were no statistically significant differences in the women’s age, BMI, parity, educational, marital status, occupation, income, and age at menarche between the two groups.

**Table 1:** Demographic and anthropometric characteristics of women with and without endometriosis


Characteristic		Case group	Control group	P value

Age (Y)^*^		31.01 ± 6.56	29.35 ± 7.00	0.13
BMI^*^^*^				0.19
	<25	50 (64.1)	50 (64.9)	
	25-29.9 (overweight)	24 (30.8)	23 (29.9)	
	≥30 (obese)	4 (5.1)	4 (5.2)	
Education^*^^*^				0.58
	Lower than university	42 (53.8)	38 (49.4)	
	University	36 (46.2)	39 (50.6)	
Age at menarche^*^^*^^*^		13.49 ± 2.38	13.35 ± 1.64	0.70
Marital status^*^^*^				0.90
	Unmarried	22 (28.2)	21 (27.3)	
	Married or cohabiting	56 (71.8)	56 (72.7)	
Parity^*^^*^				0.06
	Parous	32 (41)	42 (54.6)	
	Nulliparous	46 (59)	35 (45.5)	
Occupation^*^^*^				0.09
	Housewife	61 (78.2)	68 (88.3)	
	Employed	17 (21.8)	9 (11.7)	


*; Values are given as mean ± SD and compared using Student’s t test, **; Values are given as a number (%) and compared using Chi-squared test, ***; Values are given as mean ± SD and compared using Mann-Whitney test, and BMI; Body mass index.

Table 2 summarizes the ORs for endometriosis by daily phytoestrogen intake according to quartile of intake. We observed inverse associations between consumption of phytoestrogen (OR: 0.68; 95% CI: 0.51-0.91, P-trend=0.01) and total isoflavones (OR: 0.38; 95% CI: 0.33-0.83; P-trend=0.002) and endometriosis risk, but this difference was more related to formononetin (OR: 0.57; 95% CI: 0.27-0.97; P-trend=0.04) and glycitein (OR: 0.68; 95% CI: 0.67-0.98; P-trend=0.04). 

High consumption of lignan was associated with a lower risk of endometriosis (OR: 0.49; 95% CI: 0.46- 0.52; P-trend=0.01). Among the sub type of lignan, only secoisolariciresinol (OR: 0.54; 95% CI: 0.36-0.77; P-trend=0.01), lariciresinol (OR: 0.64; 95% CI: 0.32- 0.74, P-trend=0.02) and matairesinol (OR: 0.30; 95% CI: 0.22-0.52; P-trend=0.003) were related to reduced risk of endometriosis. The intake of coumestrol in the third quartile was associated with reduced risk of endometriosis (OR: 0.38; 95% CI: 0.15-0.96; P-trend=0.15). 

Table 3 demonstrates an association between the sub-type of phytoestrogen in each food group and risk of endometriosis. Among food groups, only isoflavin (OR: 0.48; 95% CI: 0.44-0.63), lignan (OR: 0.66; 95% CI: 0.62-0.94), coumestrol (OR: 0.64; 95% CI: 0.51-0.99) and phytoestrogen (OR: 0.46; 95% CI: 0.38-0.83) in dairy products and coumestrol in fruits (OR: 0.69; 95% CI: 0.03-0.77) were associated with endometriosis.

**Table 2:** Adjusted odds ratios (OR) of endometriosis and corresponding 95% confidence intervals (CI) according to the subtype of phytoestrogen intake


Quartile, OR (95% CI)^*^						
Phytoestrogen	1	2	3	4	OR	P trend
Total isoflavones	1.00	0.69(0.32-1.91)	0.77(0.27-1.64)	1.00(1.00-1.03)	0.38(0.33-0.83)	0.002

Genistein	1.00	0.54(0.30-1.80)	0.92(0.27-1.63)	0.92(0.26-1.54)	0.45(0.43-1.01)	0.55
Daidzein	1.00	1.00(0.99-1.06)	0.46(0.20-1.19)	0.53(0.31-1.89)	0.44(0.29-1.01)	0.18
Formononetin	1.00	0.67(0.19-1.23)	0.38(0.08-0.56)	0.35(0.18-1.17)	0.57(0.27-0.97)	0.04
Glycitein	1.00	0.71(0.11-0.81)	1.00(1.00-1.46)	0.35(0.07-0.81)	0.68(0.67-0.70)	0.04
Total lignans	1.00	0.69(0.44-1.07)	0.79(0.66-1.90)	1.00(1.00-1.01)	0.49(0.46-0.52)	0.01
Secoisolariciresinol	1.00	0.75(0.30-1.88)	0.45(0.18-1.12)	0.38(0.15-0.96)	0.54(0.36-0.77)	0.01
Pinoresinol	1.00	0.84(0.34-2.09)	0.56(0.23-1.38)	0.42(0.17-1.07)	0.49(0.40-1.02)	0.08
Lariciresinol	1.00	0.54(0.21-1.36)	0.36(0.14-0.91)	0.27(0.10-0.70)	0.64(0.32-0.74)	0.02
Matairesinol	1.00	0.38(0.16-1.33)	0.78(0.57-3.76)	0.34(0.13-0.86)	0.30(0.22-0.52)	0.003
Coumestrol	1.00	1.04(0.42-2.59)	0.77(0.31-1.88)	0.38(0.15-0.96)	0.48(0.36-1.03)	0.15
Total phytoestrogens	1.00	0.56(0.22-1.37)	0.55(0.22-1.37)	0.27(0.10-0.70)	0.68(0.51-0.91)	0.01


BMI; Body mass index and *; Odds ratio adjusted for age, energy intake, BMI, educational level, and income. Quartile 1 used as the reference category.

**Table 3:** Adjusted odds ratios (OR)* of endometriosis and corresponding 95% confidence intervals (CI) according to phytoestrogen from the dietary item


Food group	Isoflavones	Coumestrol	Lignans	Total phytoestrogens

Soy products	0.76(0.71-1.03)	0.76(0.71-1.24)	0.76(0.68-1.07)	0.74(0.71-1.03)
Legume	0.63(0.58-1.13)	0.45(0.24-0.86)	0.45(0.42-1.14)	0.62(0.59-1.08)
Nut	0.99(0.97-1.14)	0.99(0.99-1.03)	0.99(0.99-1.03)	0.99(0.98-1.01)
Dairy product	0.48(0.44-0.63)	0.64(0.51-0.99)	0.66(0.62-0.94)	0.46(0.38-0.83)
Vegetable	0.99(0.92-1.05)	0.63(0.32-1.15)	0.97(0.99-1.02)	0.97(0.96-1.02)
Fruit	0.99(0.93-1.20)	0.69(0.03-0.77)	1.01(1.00-1.01)	1.00(1.00-1.01)
Cereals and breads	0.99(0.86-1.04)	0.99(0.01-3.24)	0.94(0.87-1.01)	0.96(0.92-1.01)
Meat	0.99(0.99-1.03)	0.63(0.12-1.68)	0.99(0.72-1.15)	0.99(0.99-1.03)
Beverages, nonalcoholic	0.99(0.90-1.19)	0.98(0.66-1.53)	0.99(0.99-1.01)	0.99(0.99-1.01)


BMI; Body mass index and *; Odds ratio adjusted for age, energy intake, BMI, educational level, and income.

## Discussion

Our findings suggest that higher intake of phytoestrogen such as isoflavin, lignan, and coumestrol is associated with a reduced risk of endometriosis. All subtypes of phytoestrogen in dairy products and coumestrol in fruits were related to reduced endometriosis risk. 

Recently, some studies discussed associations between phytoestrogen and endometriosis. One of such studies (26), consumption of phytoestrogen (25 to 250 mg/ day), used for the treatment of endometriosis. Genestein inhibits the activity of tyrosine kinase, and in this way, plays an important role in growth factors signaling. While in another study (27), soy isoflavones supplementation maintained endometriosis and induced conversion of the disease to the malignant form (i.e. mixed Müllerian tumor). Moreover, using an animal model, it was shown that pharmacologic genistein, but not dietary form, helps to the maintenance of the implants (28). In a Japanese study, higher levels of urinary genistein and daidzein were related to reduced advanced endometriosis risk (29). However, to the best of our knowledge, no study has assessed possible associations between dietary phytoestrogen intake and women endometriosis risk.

Results of some studies demonstrated that dietary consumption of phytoestrogens was associated with reduced risk of endometrial, breast, colorectal and prostate cancer (30-33).

It was indicated that increased urinary excretion of phytoestrogen was associated with decreased CRP levels (13). Soy consumption is associated with a decrease in serum nitric oxide, E-selectin, interleukin-18 and CRP concentrations (34). 

Animal studies showed that ginstein has an antiproliferative effect on mammary tissue in rats exposed to prepubertal estrogens (35). Diadzein induces mitochondrial-dependent apoptosis by increasing caspase-9 activity and decreasing cyclin D expression that can affect cell cycle regulation (36). Phytoestrogens have antioxidant effects, that lead to reduced production of reactive oxygen species (ROS) (37). Genistein can suppress lymphocyte proliferation and antigen-specific immune response but enhance the cytotoxic responses mediated by NK and cytotoxic T cells and production of cytokines from T cells (38). 

Regarding the effect of phytoestrogens on inflammatory, immunological and hormonal factors, phytoestrogen consumption can reduce the risk of endometriosis. Bioavailability, absorption, and estrogenic characteristics of phytoestrogens are dependent on the compound’s bioactivity, which metabolized in to compounds by intestinal microflora (39). Also, the phytoestrogen content in diet is dependent on environmental and genetic factors for example variety, harvest, food processing, cooking and growth locations (40). Up to now, Iranian dietary phytoestrogen has not been measured. Our calculations were done based on the phytoestrogens found in the USDA food composition Table.

A limitation of this study was the problem of convincing the participants to answer many questions. Also, as it was a case-control study, the probability of selection and recall bias including under- and over-reporting of the specific food items might have affected our results.

## Conclusion

Phytoestrogens have a major impact on the level of hormones, and immune and inflammatory markers; thus, it can play an important role in the control and prevention of many diseases. Due to the inflammatory nature of endometriosis and the effect of hormones on the progression of the disease, the role of phytoestrogens consumption in the progression and regression of the disease should be assessed in future works.
